# The first polarimetric GNSS-Reflectometer instrument in space improves the SMAP mission’s sensitivity over densely vegetated areas

**DOI:** 10.1038/s41598-023-30805-7

**Published:** 2023-03-06

**Authors:** Nereida Rodriguez-Alvarez, Joan Francesc Munoz-Martin, Xavier Bosch-Lluis, Kamal Oudrhiri, Dara Entekhabi, Andreas Colliander

**Affiliations:** 1 Jet Propulsion Laboratory, California Institute of Technology, Pasadena, CA 91109 USA; 2 Massachussets Institue of Technology, Cambirdge, MA USA

**Keywords:** Hydrology, Hydrology

## Abstract

The Soil Moisture Active Passive (SMAP) mission has dramatically benefited our knowledge of the Earth’s surface processes. The SMAP mission was initially designed to provide complementary L-band measurements from a radiometer and a radar, producing geophysical measurements at a finer spatial resolution than the radiometer alone. Both instruments, sensitive to the geophysical parameters in the swath, provided independent measurements at different spatial resolutions. A few months after SMAP’s launch, the radar transmitter’s high-power amplifier suffered an anomaly, and the instrument could no longer return data. During recovery activities, the SMAP mission switched the radar receiver frequency facilitating the reception of Global Positioning System (GPS) signals scattered off the Earth’s surface, and enabling the radar to become the first spaceborne polarimetric Global Navigation Satellite System – Reflectometry (GNSS-R) instrument. With more than 7 years of continued measurements, SMAP GNSS-R data are the most extensive existing GNSS-R dataset and the only one providing GNSS-R polarimetric measurements. We demonstrate that the SMAP polarimetric GNSS-R reflectivity, derived from Stokes parameters mathematical formulation, can enhance the radiometer data over dense vegetation areas, recovering some of the original SMAP radar capability to contribute to the science products and pioneering the first polarimetric GNSS-R mission.

## Introduction

The science community has widely used radiometry and radar techniques to study the Earth’s surface^1^. While radiometers passively measure the spontaneous emission of the surfaces, radars measure the backscattering of a transmitted signal after interacting with the Earth’s surface. Based on signals of opportunity such as the Global Navigation Satellite System (GNSS) signals and also referred to as GNSS-Reflectometry, bistatic radar has also proven useful for studying the Earth’s surface by measuring the forward scattering of those GNSS signals. The main difference between backscatter and forward scatter is the direction of the reflected wave propagation. In rough surfaces or forests, backscatter is dominated by incoherent scattering and double bounce reflections. In contrast, forward scatter is dominated by coherent scattering in the specular direction, as in smooth surfaces, and does not include a strong double-bounce scattering component from vegetated areas. While different in principle, all of them, radiometer, radar, and bistatic radar, are sensitive to soil surface moisture conditions, frozen and thawed conditions of the soils, surface roughness, vegetation water content, and wetlands, among others.

The National Aeronautics and Space Administration (NASA)’s satellite mission, Soil Moisture Active Passive (SMAP), was designed to measure and produce maps of the Earth’s soil moisture (SM) and the freeze/thaw (F/T) state. The information on these geophysical parameters helps to understand terrestrial water, carbon, and energy cycles. The SMAP mission carries a radiometer and radar at L-band^2^. The maximum sensitivity to SM is achieved in the lower range of microwave frequencies from 300 MHz to 2 GHz, mostly P-band and L-band. Over the years, the scientific community concluded that the L-band range from 1.4 to 1.427 GHz was optimal. This band was primarily selected because it was already protected thanks to the astronomy community H_2_ absorption line at 1.420 GHz^3^ and the fundamental knowledge from^1,4^. Furthermore, the atmosphere effect in that band is nearly non-existent, providing sensitivity to SM with all-weather coverage, and the vegetation layer effect is limited, allowing observations of the soil under it (e.g.^5,6^). Consequently, the SMAP radiometer was designed at 1.413 GHz^7^, and the SMAP radar was designed slightly off at 1.26 GHz^8^, at a reserved band for radar. SMAP was launched on January 31, 2015. On July 7, 2015, a few months after SMAP’s launch, the radar transmitter suffered an anomaly and prevented it from its nominal operation, ceasing the collection of the originally intended radar dataset^9^.

**Figure 1:**
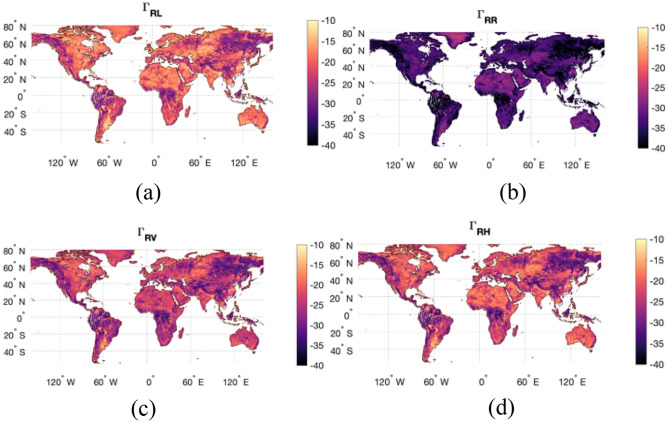
Contrast between bistatic radar signatures of an RHCP transmitted signal scattering from the Earth’s surface and measured with (**a**) an LHCP antenna, (**b**) an RHCP antenna, (**c**) an H polarization antenna and (**d**) a V polarization antenna. Maps were created using have been created using Matlab R2021b (version 9.11.0.1809720, URL: https://www.mathworks.com/products/new_products/release2021b.html).

Shortly after the radar transmitter anomaly occurred, the mission decided to switch the radar receiver bandpass filter from the original frequency of 1.26 GHz to 1.22742 GHz frequency. By doing that, the mission enabled the reception of Global Positioning System (GPS) L2C signals as they scatter off the Earth’s surface, converting the SMAP radar receiver into an opportunistic reflectometer (SMAP-Reflectometer or SMAP-R). In 2015, this change resulted in the second mission in the world that could conduct GNSS-Reflectometry (or GNSS-R). The co-existing TechDemoSat-1 (TDS-1^10^) mission also carried a reflectometer designed to work at the GPS L1 band. Later, the third GNSS-R mission was launched, the CYclone Global Navigation Satellite System (CYGNSS^11,12^) mission, specifically designed for the application of hurricane detection and monitoring. For a while, the three missions co-existed, but TDS-1 was decommissioned in 2018. In addition to the flagship GNSS-R missions mentioned, a few new concepts are being formulated to include polarimetric capabilities as the next steps in the reflectometry field, e.g. a few CubeSat missions as well as the planned polarimetric GNSS-R mission HydroGNSS^13^ from the European Space Agency (ESA). When this manuscript was written, the only major satellite missions providing open-access reflectometry data were CYGNSS and SMAP. CYGNSS measures the reflected form of the transmitted Right Hand Circularly Polarized (RHCP) GPS signals in a standard configuration, i.e. using Left Hand Circularly Polarized (LHCP) antenna to capture the strongest scattering resulting from the reversed sense of circularity of the coherent surface bounces. SMAP-Reflectometry unconventionally measures the reflected GPS signals in both horizontal (H) and vertical (V) linear polarizations. To show the strength of bistatic radar signatures at different polarizations, Fig. 1 shows the contrast between the reflectivity measured at LHCP, RHCP, H and V polarizations from a RHCP transmitted signal, i.e. ${\Gamma }_{RL}$, ${\Gamma }_{RR}$, ${\Gamma }_{RV}$, and ${\Gamma }_{RH}$ respectively, derived from SMAP-Reflectometer data at 40° incident angle.

Note that the polarimetric formulation is defined through hybrid compact polarimetry (HCP) Synthetic Aperture Radar (SAR) theory^4,14–18^ by computing the four Stokes parameters from the received RHCP-H and RHCP-V electromagnetic fields, as defined in the “Methods” section, and the complete SMAP-Reflectometry mathematical formulation is described in detail in^19^. From Fig. 1, while ${\Gamma }_{RL}$ captures the strongest signatures, ${\Gamma }_{RR}$ shows the weakest returns. The best choice for non-polarimetric GNSS-R missions is LHCP antennas, but a GNSS-R polarimetric mission with a low-gain antenna would greatly benefit from the use of linear polarizations to maximize the probability to detect the two orthogonal polarization components^20^. SMAP-Reflectometry gives a unique opportunity to study polarimetric GNSS-R sensitivities.

In this manuscript, we demonstrate that the information obtained from this unique polarimetric GNSS-R dataset can enhance the SMAP radiometer data over dense vegetation areas. Similarly to what was envisioned to achieve an SM product resulting from active/passive measurements from the SMAP radar/radiometer instruments, the SMAP-Reflectometer acquired data can help the radiometer measurements improve SM estimates over vegetated areas. SMAP-Reflectometer measurements used in our analysis can also be used to enhance the coarse brightness temperature measurements from the radiometer into finer gridded brightness temperature maps, similar to what is done in the 9 km SM product through Backus-Gilbert interpolation^21,22^, but using SMAP-Reflectometer data (forward scattering information) to perform the interpolation. Our approach is tested by performing SM retrievals using the radiometer ${T}_{B}$ and the ${T}_{B}$ enhanced with SMAP-Reflectometer data and then comparing them to ground stations from core validation sites (CVS)^23^. This contribution not only returns some of the original SMAP radar capability to contribute to the mission’s science products but also demonstrates the scientific value of SMAP-Reflectometer as the first fully polarimetric GNSS-R instrument in space, establishing SMAP as the first fully polarimetric GNSS-R mission^24^.

## SMAP’s mission data

**Figure 2:**
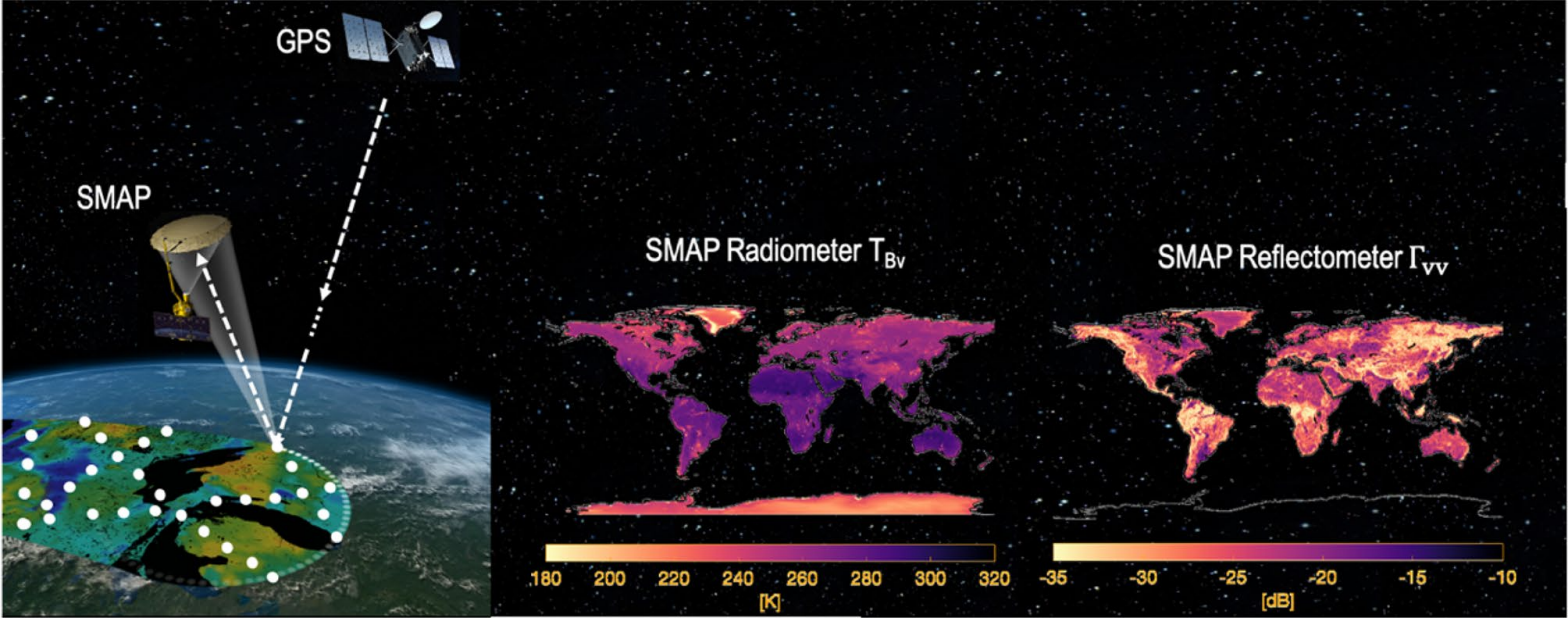
SMAP-Reflectometry—Conceptual sketch of SMAP radiometer and SMAP-Reflectometer measurements while scanning the Earth’s surface, including brightness temperature (${T}_{Bv}$) measurements from SMAP radiometer (top-left) and reflectivity (${\Gamma }_{vv}$) measurements computed from SMAP-Reflectometer data (bottom-left), gridded at 36 km and 9 km, respectively, for 1 year of data in 2018. Note that ${\Gamma }_{vv}$ is estimated from the Stokes parameters computed from SMAP-Reflectometer data by assuming negligible cross-polarization. This is further explained in “Methods” section. SMAP Radiometer and SMAP Reflectometer satellite data maps have been created using Matlab R2021b (version 9.11.0.1809720, https://www.mathworks.com/products/new_products/release2021b.html). The Artist’ rendering is modified using Microsoft PowerPoint for Mac version 16.69 from the original image (Credit to NASA/JPL-Caltech) freely available in (https://www.jpl.nasa.gov/images/pia19133-soil-moisture-active-passive-satellite).

Even though SMAP-Reflectometer is a pioneer in the GNSS-R polarimetry field, it has never been perceived as a mission of reference. Figure 2 shows a conceptual sketch of the SMAP satellite and the measurements collected by the suite of instruments for 1 year of data. Figure 2 demonstrates features that suggest to the naked eye that the reflectivity at vertical polarization ${\Gamma }_{vv}$ obtained from SMAP-Reflectometer data are sensitive to the geophysical parameters of the land, e.g. SM, how much water is in the plants, i.e. vegetation optical depth (VOD), the surface roughness, the presence of standing water, snow, ice, and the F/T state, as one can easily recognize the different landscapes. Similarly, SMAP-Reflectometer-derived reflectivity at horizontal polarization (${\Gamma }_{hh}$) shows similar features (not shown). Note that both ${\Gamma }_{vv}$ and ${\Gamma }_{hh}$ are estimated from the Stokes parameters computed from SMAP-Reflectometer data by assuming negligible cross-polarization, as shown in the “Methods” section. The combined information of the two polarimetric signatures ${\Gamma }_{hh}$ and ${\Gamma }_{vv}$ is sensitive to the presence of vegetation and is, therefore, a valuable asset that can help enhance SMAP radiometer brightness temperatures ${(T}_{B})$ to produce better SM estimates over vegetated areas. Equally, SMAP-Reflectometer data are particularly sensitive to the frozen and thaw state^25,26^ of different landscapes, and the measurements can help produce enhanced F/T products for the SMAP mission. In this paper, we are not targeting the development of a standard retrieval algorithm for any specific science application. Still, we are using the SM estimation case as a practical example to highlight the relevance of the dataset collected by the SMAP-Reflectometer.

## SMAP mission soil moisture accuracy

The SMAP mission, launched on January 31, 2015, by combining the measurements of the radiometer and the radar for the first time, planned to provide SM and F/T products with increased resolution and accuracy. When the radar malfunctioned, the mission had collected approximately 2.5 months of data from the radar. The combined active/passive SMAP high-resolution SM product derived from several alternative algorithms was evaluated against in situ data from CVS and sparse networks^27,28^. From the different alternative disaggregation algorithms evaluated, the baseline algorithm, i.e. disaggregating the radiometer brightness temperature to 9 km and retrieving the soil moisture at 9 km, performed best, obtaining an overall unbiased root-mean-square-difference (ubRMSD) of 0.039 m^3^/m^3^ meeting the 0.04 m^3^/m^3^ soil moisture accuracy requirement^27^.

In an effort to recover the SMAP mission’s originally designed active/passive capability, a new product was created, which combined the SMAP radiometer measurements with the Copernicus Sentinel-1A/B C-band backscatter data^29^. In a recent study, the different SMAP soil moisture products were validated using data from CVS and sparse networks^30^. When compared to CVS, the Dual-Channel Algorithm (DCA) exhibited good performance over grassland (0.031 m^3^/m^3^) and cropland (0.039 m^3^/m^3^) sites, and were slightly over the requirement for crop/natural mixed sites (0.047 m^3^/m^3^)^30^. Compared to sparse networks, DCA results showed that only savannas, barren/sparse, and evergreen needleleaf forests met SM mission requirements^30^. When using the merged SMAP/Sentinel-1 product, the results improve over croplands (0.030 m^3^/m^3^) when analyzed over CVS. For another agricultural location, the SMAP/Sentinel-1 active–passive soil moisture product showed a ubRMSD between 0.017 and 0.051 m^3^/m^3^ during the non-growing season but a ubRMSD between 0.089 and 0.104 m^3^/m^3^ for the growing season^31^. In 2021 Sentinel-1B stopped operating, which reduced the revisit time for the combined SMAP/Sentinel-1 product.

## The added value of SMAP-reflectometry

The SMAP mission has given the GNSS reflectometry science community an invaluable opportunity that invites further exploration. The dataset was not officially processed or provided as a SMAP product for years, primarily because of its processing and calibration complexity. A small group of researchers, including the leading authors in this paper, initially made parallel efforts over the years^32–35^. Still, the investigations only processed a minimal amount of data to show the basic capability of the dataset, and did not provide the formal mathematical and electromagnetic basis of the polarimetric dataset collected from the SMAP-Reflectometer.

The authors have developed a processor for the dataset collected from the SMAP-Reflectometer to provide a calibrated product and enable science applications. The SMAP mission was never designed to have a reflectometer instrument; however, the availability of the radar receiver in the suitable band and the signals transmitted by the GPS satellites enabled the mission to have this capability. The high-gain antenna offers an unprecedented signal-to-noise ratio no other reflectometry mission has observed. However, its conical scan and narrow footprint restrict the data collection to precise relative geometries between the satellite transmitting the signal and the SMAP antenna pointing, which results in limited coverage over short periods. At the same time, because of the narrow footprint at geometries around 40° incident angle (± 3°), measurements are ideal for collecting polarimetric information of the scenes at V and H polarizations.

**Figure 3:**
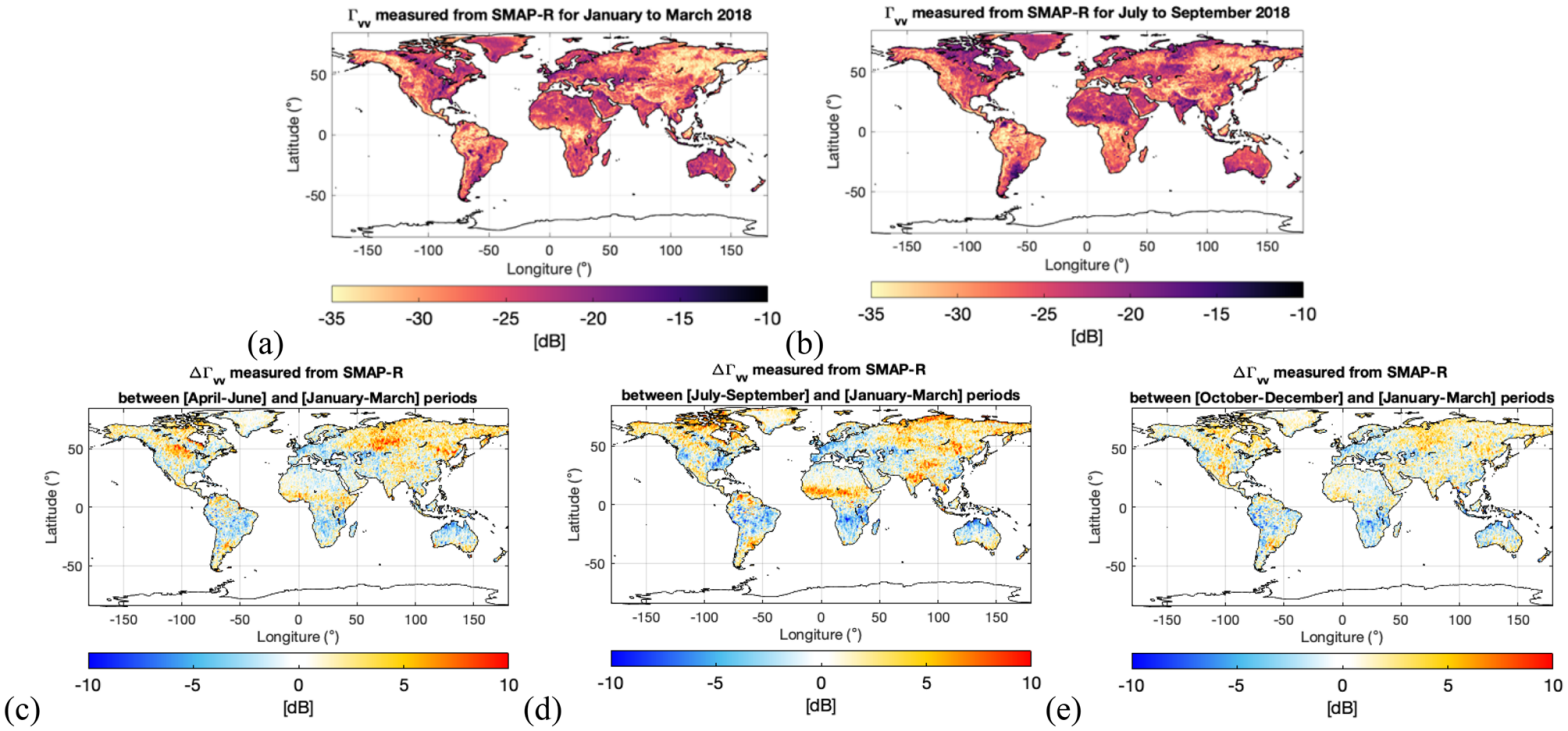
A unique view of the Earth’s surface achieved by SMAP GNSS-R dataset gridded 9 km during the year 2018: (**a**) Reflectivity at v-polarization (${\Gamma }_{vv}$) computed from the SMAP GNSS-R Dataset for the first quarter (Q1: January to March), (**b**) ${\Gamma }_{vv}$ computed from the SMAP GNSS-R Dataset for the third quarter (Q3: July to September); and difference ${\Delta\Gamma }_{vv}$ observed between Q1 and (**c**) Q2: April to June, (**d**) Q3: July to September; and (**e**) Q4: October to December. All figures made with Matlab R2021b (version 9.11.0.1809720, URL: https://www.mathworks.com/products/new_products/release2021b.html).

The SMAP-Reflectometer unconventionally measures, at linear polarizations (H and V), a signal transmitted at right-hand circular polarization (RHCP) and then scattered from the Earth’s surface at a mix of polarization, depending on the scattering surface itself. This is known in the SAR field as hybrid compact polarimetry (HCP)^4,14–18^, but for the specific case of reflectometry, this is a full polarimetric GNSS reflectometer^19,24^. Hence, SMAP-R is the first-ever full-polarimetric GNSS reflectometer in space, as it is able to receive the full polarimetric portrait of the scene^24^. It offers the unique capability to retrieve the Stokes parameters of the reflected wave, giving an unprecedented view of the Earth. In^19^, the mathematical concept basis for the SMAP-R GNSS polarimetric reflectometer and the Stokes parameter computation was presented, including the proper calibration of the dataset collected from the SMAP-Reflectometer and validation of the methodology. The maps in Fig. 3 show the unique view of the Earth offered by the reflectivity at vertical polarization ${\Gamma }_{vv}$ and the subsequent seasonal changes. ${\Gamma }_{vv}$ is obtained from applying Eqs. (1) and (2), in the “Method” section, following^19^ and a similar theory in^4,14–18^. Data in Fig. 3 maps correspond to 2018 and has been merged into a 9-km grid using particular months to capture seasonality. Missing data have been completed using natural neighbor interpolation^36^.

Figure 3d shows changes in the reflectivity at v-polarization ${\Gamma }_{vv}$ between the first quarter of the year 2018 (Q1: January to March) and the ${\Gamma }_{vv}$ for the third quarter of the year 2018 (Q3: July to September). Figure 3c shows the changes between Q1 and Q2 (April and June), and Fig. 3e shows the changes between Q1 and Q4 (October and December). The differences between Q3 and Q1 are the most evident, with increased ${\Gamma }_{vv}$ values over Alaska, due to the thawing state during Q3 and the frozen state in Q1; also same applies to Russian Siberia. Additionally, we can see an increase in ${\Gamma }_{vv}$ over central Africa (tropical region) due to the SM increase from the amount of rain during the wet season. The African tropical region’s wet season, which expands from May to August, peaks in July. The differences between Q2 and Q1and Q4 and Q1 show the transitions between the summer and winter seasons. A very distinctive signature is observed between Q2 and Q1 for the United States Corn Belt, clearly showing an increase in ${\Gamma }_{vv}$ for the transition from the winter to the start of the growing season, when the vegetation starts to build up a higher water content (a higher VOD), and then in Q3 as crops dry and are being harvested, the signature fades out. The sensitivity of GNSS-R signals to the vegetation layer (biomass, VOD) has been proven in previous studies, e.g.^35,37,38^.

**Figure 4:**
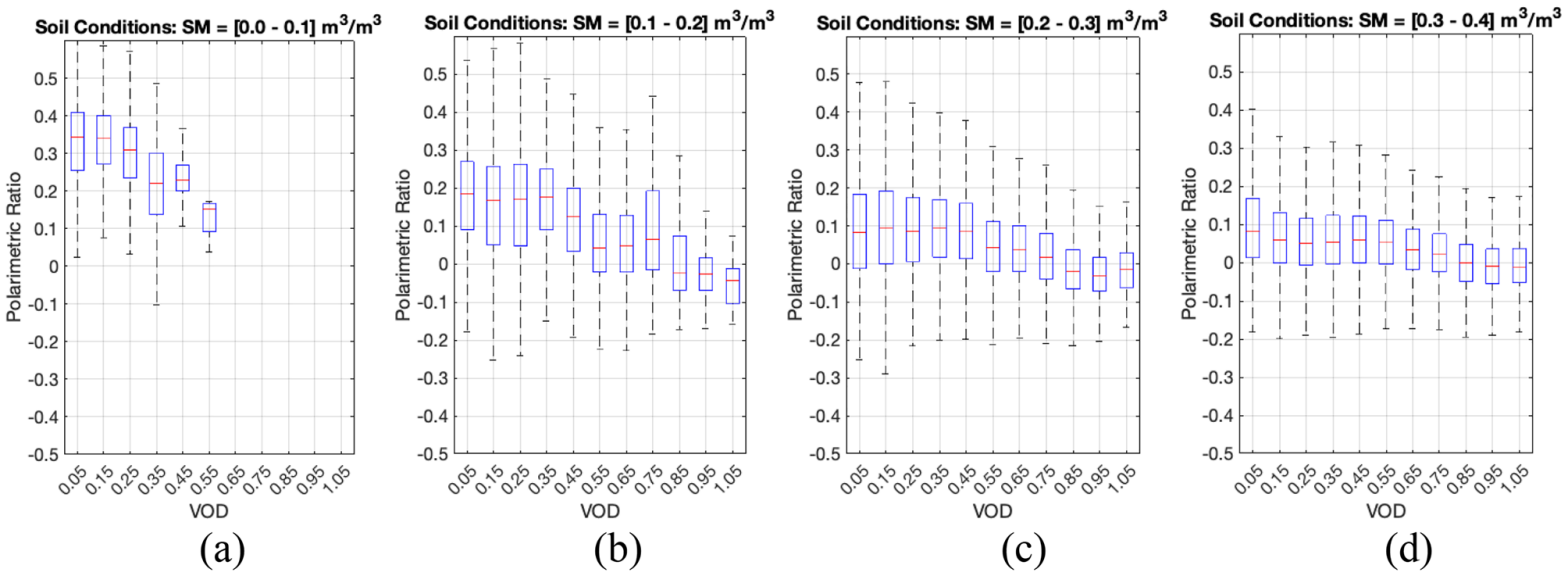
Sensitivity of SMAP GNSS-R dataset gridded 9 km to VOD variations of 0.1 for a range of SM: (**a**) [0.0 – 0.1] m^3^/m^3^, (**b**) [0.1 – 0.2] m^3^/m^3^, (**c**) [0.2 – 0.3] m^3^/m^3^, and (**d**) [0.3 – 0.4] m^3^/m^3^. All figures made with Matlab R2021b (version 9.11.0.1809720, URL: https://www.mathworks.com/products/new_products/release2021b.html).

The dataset collected from the SMAP-Reflectometer provides the GNSS-R community with a polarimetric GNSS-R implementation that may not have been tested if it were not for SMAP. The dataset will provide, in return, the SMAP mission with an opportunity to recover some of the capabilities lost when the radar transmitter malfunctioned. The data collected by the SMAP radar receiver can facilitate a similar role to that of the SMAP radar over vegetated areas. Over short periods, the SMAP-Reflectometer measurements are sparse, and the benefit of using these measurements to improve sample-to-sample SMAP radiometer brightness temperatures may be limited. Still, over seasonal time periods, SMAP-Reflectometer offers very useful information over areas where vegetation does not undergo a strong seasonal cycle, such as forests, and where SMAP radiometer SM products tend to underperform. The sensitivity of SMAP-Reflectometry data to SM and VOD is shown in Fig. 4 through the computation of the normalized polarimetric ratio, i.e. ${\mathrm{PR}=(\Gamma }_{hh}- {\Gamma }_{vv})/({\Gamma }_{hh}+ {\Gamma }_{vv})$.

Figure 4 shows the polarimetric sensitivity of SMAP-Reflectometry signals to VOD, obtained from SMAP ancillliary datasets, for a given range of SM. As the SM increases, the sensitivity to VOD is reduced because the SM contribution in the signatures increases and diminishes the sensitivity to VOD changes. The variations observed in the plots can be approximated as the normalized polarimetric ratio range (${\Delta }_{PR}$) within a complete VOD range [0–1.05] of ${\Delta }_{PR}$ = 0.35, 0.19, 0.09, and 0.08, respectively, for the ranges in Fig. 4a to d. As a possible application for these data, one could take the SM estimates from the SMAP mission as accurate values and generate monthly or seasonal VOD maps following a set of curves like the ones in Fig. 4. This static information can then be used to improve the daily SM retrievals from the SMAP radiometer, generating a new radiometer/reflectometer product where daily SMAP radiometer data are improved over vegetated areas through monthly/seasonal measurements of SMAP-Reflectometer over the same areas.

## Returning capabilities

The SMAP mission had planned to employ the backscatter p-polarimetric (with $p=v,h$) measurements (${\sigma }_{pp}$) from the SMAP radar to disaggregate the SMAP radiometer brightness temperatures (${T}_{Bp}$). The radar data were obtained at 1 km spatial resolution, while the radiometer data were obtained at 36 km spatial resolution. The combination of these two datasets was expected to produce soil moisture products at 3 km and 9 km spatial resolutions. When the radar stopped working, the SMAP mission applied a Backus-Gilbert interpolation^21,22^ to regrid the SMAP brightness temperatures onto a 9-km grid, but without improving the spatial resolution. Analogously, we have developed an algorithm that uses the SMAP-Reflectometer measurements (${\Gamma }_{pp}$) to interpolate the SMAP radiometer measurements onto a 9-km grid. This aided interpolation is expected to improve SM estimates thanks to the polarimetric information contained in the enhanced ${T}_{Bp}$, particularly in the presence of vegetation. As presented in^34^, the average SMAP-Reflectometer spatial resolution for wetlands is 2.8. km, for arid land 5.3 km, for medium–low vegetation 12.1 km, and for very dense vegetation 26.6 km. Taking into account the distribution of land cover around the globe, the mean SMAP-Reflectometer pixels is 9 km, and consequently, the SMAP-Reflectometer data were gridded on a 9-km grid. Note that 9 km is not the true spatial resolution of SMAP-Reflectometer for each sample, but the information contained in the mosaic maps can, in the mean, provide sub-36 km information valuable to SMAP mission products. When the SMAP-Reflectometer data are accumulated over 1 month and then re-gridded to a fixed 9 km pixel grid using a natural neighbor interpolation, daily variability is smoothened away, and the remaining features correspond to more static geophysical parameters, such as VOD or roughness. Using monthly SMAP-Reflectometer data to interpolate daily SMAP radiometer data into a 9-km product helps improve the interpolation strategy aided by an independent, physically meaningful source of information.

**Figure 5:**
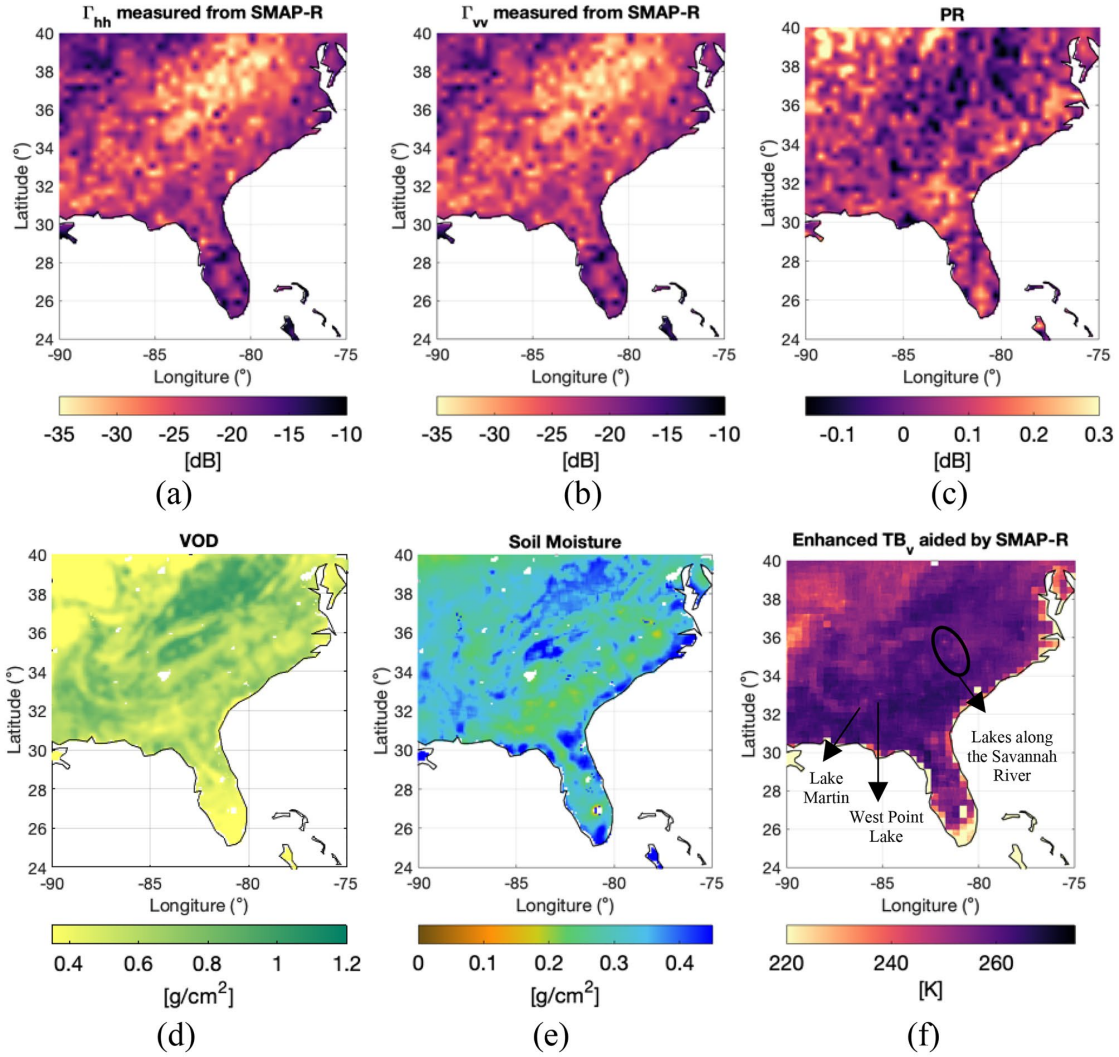
Aided-interpolation of SMAP radiometer data using SMAP-Reflectometer data over the study region: (**a**) SMAP-Reflectometer ${\Gamma }_{hh}$ at 9 km, (**b**) SMAP-Reflectometer ${\Gamma }_{vv}$ at 9 km, (**c**) Polarimetric ratio computed as ${\mathrm{PR}=(\Gamma }_{hh}- {\Gamma }_{vv})/\left({\Gamma }_{hh}+ {\Gamma }_{vv}\right)$, (**d**) VOD of the region, (**e**) soil moisture of the region, and (**f**) resulting SMAP ${T}_{Bv}$ at 9 km. All figures made with Matlab R2021b (version 9.11.0.1809720, URL: https://www.mathworks.com/products/new_products/release2021b.html).

Therefore, we have developed a scheme in which we enhance original SMAP brightness temperatures by using SMAP-Reflectometer data gridded at 9 km to interpolate the information of the radiometer at 36 km spatial resolution into a smaller grid in which the data used for interpolation are sensitive to a mean SM over the month, vegetation optical depth and roughness through a mechanism (forward scattering) independent from that of the radiometer (spontaneous emission). Figure 5 shows the data over a specific area within the Continental U.S. (CONUS) for the reflectometer, the mean SM and mean VOD in that same region and the 9 km enhanced ${T}_{Bv}$ resulting from applying the SMAP-Reflectometer ${\Gamma }_{vv}$ aided-interpolation to the 36 km SMAP radiometer ${T}_{Bv}$ data . Results are shown at v-pol, but it is equivalent at h-pol.

We focus on one particular region, encompassing Florida, South Carolina, North Carolina, Tennessee, Alabama, and Georgia. Within this region, there are available CVS data that we will use to evaluate the differences between the SM retrieved from the original ${T}_{Bv}$ and the interpolated ${T}_{Bv}$. The selected CVS (Little River, Georgia) allows testing the usefulness of using SMAP-Reflectometer for aiding the interpolation over vegetated areas where the SM product has shown higher errors^30^. Plots in Fig. 5a and b provides the SMAP-Reflectometer ${\Gamma }_{vv}$ and ${\Gamma }_{hh}$ at 9 km, respectively$.$ Fig. 5c corresponds to the polarimetric ratio $\mathrm{PR}$, computed from ${\Gamma }_{vv}$ and ${\Gamma }_{hh}$ data. Figure 5d provides the VOD of the region and Fig. 5e provides the SM of the region. Finally, Fig. 5f provides the interpolated ${T}_{Bv}$ obtained from the algorithm explained in the “Methods” section. In particular, Fig. 5f demonstrates bright spots matched to lakes, such as West Point Lake, Lake Martin, and Lakes along the Savannah River, as a straightforward display of the ability of the SMAP-Reflectometer data to bring in features of the surface. The water presence information, the increased granularity linked to roughness and the variety of landscape surfaces are contained in SMAP-Reflectometer data and are incorporated into the interpolated ${T}_{Bv}$. The same methodology is applied to the h-polarized ${T}_{Bh}$ with ${\Gamma }_{hh}$, obtaining an aided-interpolated ${T}_{Bh}$ (not shown). Note how low PR in Fig. 5c correlates with higher VOD in Fig. 5d, and vice versa, as shown in Fig. 4, and confirming that ${\Gamma }_{vv}$ and ${\Gamma }_{hh}$ contain VOD information.

**Figure 6:**
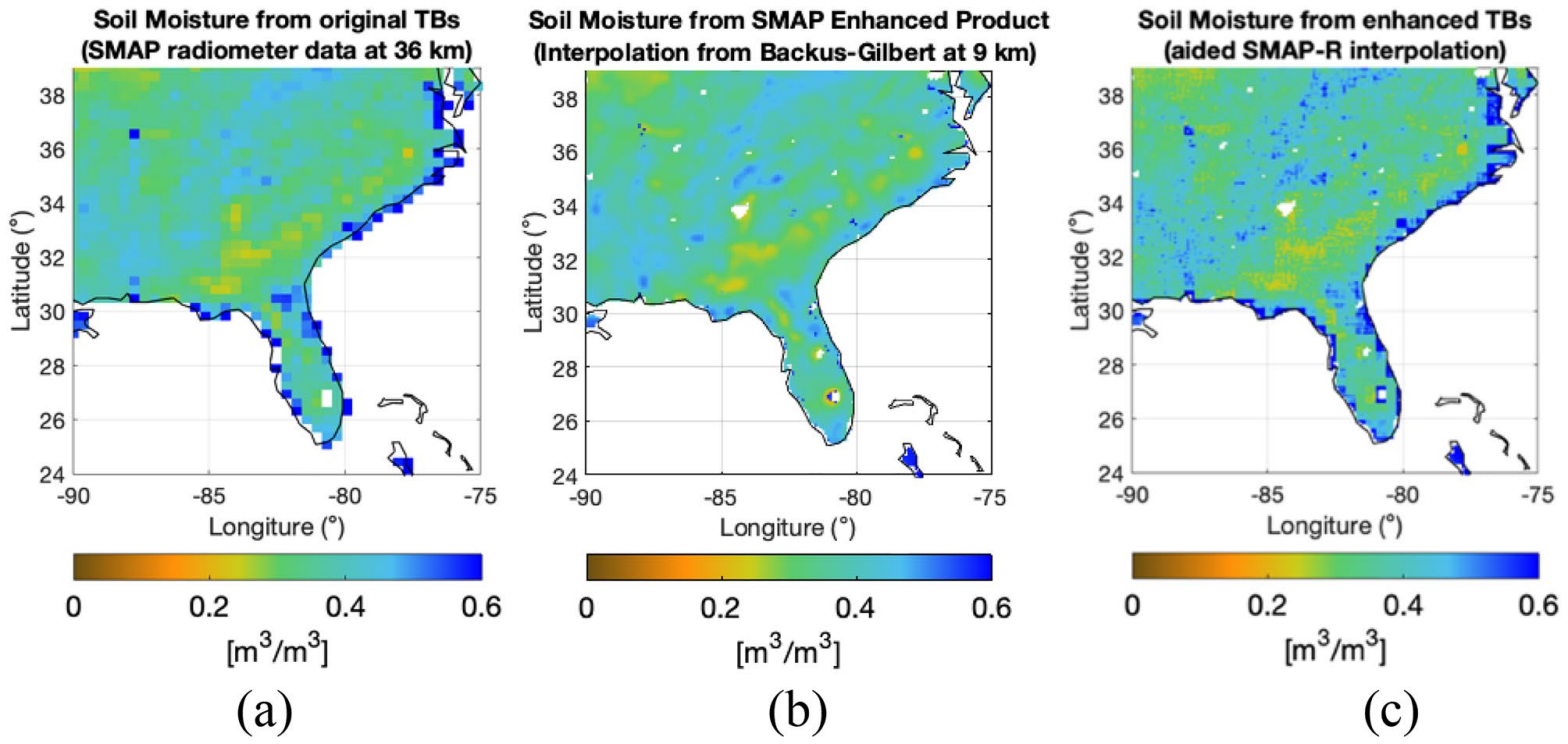
SM retrieved over Florida's region using (**a**) original ${T}_{Bv}$ from SMAP radiometer alone at 36 km, (**b**) enhanced ${T}_{Bv}$ through Backus-Gilbert, and (**c**) aided-interpolated ${T}_{Bv}$ resulting from combining SMAP radiometer and SMAP-Reflectometer. All figures made with Matlab R2021b. (version 9.11.0.1809720, URL: https://www.mathworks.com/products/new_products/release2021b.html).

**Figure 7:**
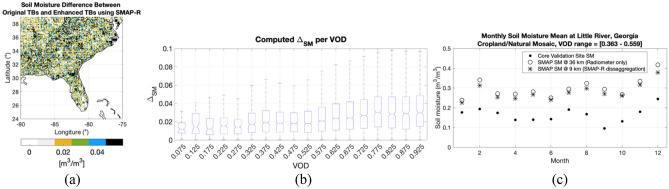
Soil Moisture Analysis: (**a**) Mean difference between mean SM from SMAP original TBs and SM from enhanced SMAP TBs using SMAP-R; (**b**) box plot of the mean and std of the differences between SM products as a function of VOD; and (**c**) comparison of 2018 monthly soil moisture (SM) means for the Core Validation Site (CVS) in Little River, Georgia, with VOD values ranging from 0.363 to 0.559 through the year. All figures made with Matlab R2021b (version 9.11.0.1809720, URL: https://www.mathworks.com/products/new_products/release2021b.html).

We then use an artificial neural network (ANN) trained model to mimic the DCA output. The SM-ANN model is trained from the mission data, i.e. targeting SM from ${T}_{Bh}$, ${T}_{Bv}$, land surface temperature and VOD. Consequently, we obtain the SM product by ingesting the original ${T}_{Bp}$ values, and the enhanced SM product by ingesting the enhanced ${T}_{Bp}$ values. Figure 6 provides the SM maps for the region under study. The SM images in Fig. 6 are similar, but in detail, some differences are introduced by the granularity of the SMAP-Reflectometer measurements. Note the river system captured over Florida’s region in Fig. 6c when the SMAP-Reflectometer data are used, in comparison to Fig. 6a or b, where the SM product from the original ${T}_{B}$ and the SM product enhanced through Backus-Gilbert are not able to see as much detail. Figure 7 analyzes the differences between products and the differences in the CVS values every month.

Figure 7a shows the difference between the mean SM values for 2018 of the two products: SM from SMAP original ${T}_{B}$ s and SM from enhanced SMAP ${T}_{B}$ s using SMAP-R. By comparing Figs. 7a and 5d, the majority of blue and black values (${\Delta }_{SM}>0.04$) are observed in areas with higher VOD, and white and grey values (${\Delta }_{SM}<0.02$) are observed in areas with lower VOD. This is also shown in Fig. 7b, with a trend whereas VOD increases, the ${\Delta }_{SM}$ is larger. Figure 7c shows the monthly SM means obtained from our SM-ANN model for CVS Little River in Georgia, representing an area characterized by cropland and natural mosaic with a VOD ranging from 0.36 to 0.56 throughout 2018. VOD values are obtained from SMAP ancillary dataset provided along the SMAP L3 Radiometer Global Daily 36 km EASE-Grid Soil Moisture version 8 (SPL3SMP^39^). As shown in Fig. 7, the monthly means of the SM product derived from original ${T}_{B}$ s are consistently higher than the monthly means of the SM product from the aided-interpolated ${T}_{B}$ s compared to the CVS SM value, with 0.05 differences in some of the months for both sites. Finally, Table 1 summarizes the SM yearly mean ubRMSD for each product at sites with VOD values over 0.2 within the year. Table 1 shows that the error associated with the SM retrieved from the interpolated ${T}_{B}s$ at 9 km aided by SMAP-Reflectometer consistently provides a lower error, compared to the CVS provided values. The added information from SMAP-Reflectometer brings these errors down consistently as new uncorrelated information from the reflectometer, i.e. the scattering of the GPS signal over the Earth’s surface landscape, complements the radiometer-measured spontaneous emission from the surface and vegetated layers.

**Table 1:** Yearly mean ubRMSD, bias, and Pearson correlation coefficient I for the soil moisture (SM) estimates compared to in-situ measurements at different CVS.

Core validation site|metric	SM performance @ 36 km grid			SM performance @ 9 km grid		
	(Radiometer only)			(Aided SMAP-reflectometer interpolation)		
	ubRMSD (m^3^/m^3^)	Bias (m^3^/m^3^)	R	ubRMSD (m^3^/m^3^)	Bias (m^3^/m^3^)	R
Little river, GA, US (VOD range = [0.363 to 0.559])	0.035	0.134	0.68	0.028	0.094	0.73
Tonzi ranch, CA, US (VOD range = [0.247—0.31])	0.041	0.078	0.92	0.033	0.086	0.93
AMMA-Catch-Benin, Benin, (VOD range = [0.281—0.36])	0.032	0.081	0.91	0.026	0.061	0.94
HOBE, Denmark (VOD range = [0.285—0.494])	0.038	0.052	0.81	0.034	0.035	0.84
Carman, Canada, (VOD range = [0.087—0.32])	0.040	0.069	0.48	0.037	0.056	0.51
Kenaston, Canada, (VOD range = [0.015—0.312])	0.017	0.710	0.93	0.014	0.69	0.95
South Fork, Iowa, (VOD range = [0.014—0.467])	0.037	0.023	0.85	0.030	0.021	0.94

Comparison is evaluated for the two different SM products obtained from ingesting radiometer only data and radiometer data interpolated with SMAP-Reflectometer at 9 km into the SM-ANN model.

## Methods

To obtain the reflectivity from SMAP-Reflectometer, we follow the rationale in^19^, where the full Stokes parameters are computed from the GPS signals transmitted at Right Hand Circular Polarization (RHCP, or R) which reflect of the Earth’s surface and are measured with SMAP-Reflectometer at H-pol and V-pol. This is summarized in Eq. (1):

1 $$\begin{gathered} S_{0} = {\text{~}}\, < |E_{{RH}} \left. \right|^{2} > \,\, + \,\, < |E_{{RV}} \left. \right|^{2} > , \hfill \\ S_{1} = \,\, < |E_{{RH}} \left. \right|^{2} > - < |E_{{RV}} \left. \right|^{2} > , \hfill \\ S_{2} = 2 < Re\{ E_{{RH}} E_{{RV}}^{{\text{*}}} \} > , \hfill \\ S_{3} = 2 < Im\{ E_{{RH}} E_{{RV}}^{{\text{*}}} \} > . \hfill \\ \end{gathered}$$

Following previous studies on HCP Synthetic Aperture Radar (SAR)^4,14–18^ if cross-polarization is considered negligible, i.e. ${\Gamma }_{HV }\sim {\Gamma }_{VH}$ are at least an order of magnitude smaller than ${\Gamma }_{HH}$ and ${\Gamma }_{VV}$, which is the case for L-band forward scatter radar^40^, then the reflectivity can be approximated as:

2 $$\begin{gathered} {\Gamma }_{HH} \cong { }\frac{{S_{0} + S_{1} }}{2}, \hfill \\ {\Gamma }_{VV} \cong { }\frac{{S_{0} - S_{1} }}{2}. \hfill \\ \end{gathered}$$

Thanks to the SMAP orbit at 6 AM/6 PM, the ionospheric total electron content (TEC) due to Sun radiation is minimal^41^. For this study, we consider a maximum Faraday rotation of 10°^42^. In this case, the impact in the normalized polarization ratio ($PR={S}_{1}/{S}_{0}$) is given by $({S}_{1}\cdot \mathrm{cos}(2{\theta }_{F})-{\mathrm{S}}_{2}\cdot \mathrm{sin}(2{\theta }_{F}))$. For the worst-case scenario, i.e. having a total two-way Faraday rotation of 10°, and ${\mathrm{S}}_{2}\sim 0$, the measurement is underestimated by 6%^42^. Within this study, the Faraday rotation is assimilated as part of the error in our measurement. Further studies will be performed to study the impact of the Faraday rotation on full polarimetric GNSS-R signals and its corresponding calibration.

The aided-interpolation method is inspired by the disaggregation approach developed for the SMAP radar backscatter measurements^2^. The SMAP mission developed a series of equations leading to a linear relationship between the two instrument measurements^2^: ${T}_{Bp}= \alpha + \beta {\sigma }_{pp}$, with $\alpha$ and $\beta$ estimated from the measurements as a best linear fit, and ${T}_{Bp}$ the radiometer brightness temperature at $p$ polarization (being $p=v,h$) and ${\sigma }_{pp}$ the radar energy backscatter co-pol measurement at $p$ polarization. To estimate $\alpha$ and $\beta$, the radar data were aggregated into the coarser resolution of 36 km. Then the disaggregation is conducted as explained in^2^ through eq. (19) to eq. (26), and summarized here in Eq. (3):

3 $${T}_{Bv}\left({M}_{j}\right)= {T}_{Bv}\left(C\right)+ \beta \left(C\right)\cdot \left\{\left[{\sigma }_{vv}\left({M}_{j}\right)-{\sigma }_{vv}\left(C\right)\right]+\zeta \cdot \left[{\sigma }_{vh}\left(C\right)-{\sigma }_{vh}\left({M}_{j}\right)\right]\right\},$$

where ${M}_{j}$ is the medium resolution of grid cell $j$ and $C$ is the coarse resolution cell of 36 km, with $\alpha \left({M}_{j}\right)= \alpha \left(C\right)$, disappearing from the equation, and $\beta \left({M}_{j}\right)= \beta \left(C\right)$ simplifying the expression. The parameter $\zeta$ is the sensitivity and is computed as $\zeta ={\left[\frac{\partial {\Gamma }_{vv}\left({M}_{j}\right)}{\partial {\Gamma }_{vh}\left({M}_{j}\right)}\right]}_{C}$, which is specific to the particular grid cell and the particular season for grid cell. $\zeta$ is estimated based on collecting the co-polarization and cross-polarization radar backscatter data within each grid cell.

Analog to the process applied to the backscatter information from the radar in^2^, we assume that there exist a linear relationship between forward scatter and brightness temperature, i.e. ${T}_{Bv}\left(\mathrm{C}\right)= {\alpha }^{\mathrm{^{\prime}}}+ {\beta }^{\mathrm{^{\prime}}}\left(C\right){\Gamma }_{vv}\left(C\right)$ for each pixel where $\alpha \mathrm{^{\prime}}$ and $\beta \mathrm{^{\prime}}$ are different from the $\alpha$ and $\beta$ used for the radar and can also be estimated from the SMAP radiometer and the SMAP reflectometer measurements as a best linear fit. Then following same rationale as in^2^ through Eq. (19) to Eq. (26), but using ${T}_{Bv}\left(\mathrm{C}\right)= {\alpha }^{\mathrm{^{\prime}}}+ {\beta }^{\mathrm{^{\prime}}}\left(C\right){\Gamma }_{vv}\left(C\right)$ as starting point we can write an analogous equation to Eq. (3) for the SMAP-Reflectometry ${\Gamma }_{vv}$ as:

4 $${T}_{Bv}\left({M}_{j}\right)= {T}_{Bv}\left(C\right)+ {\beta }^{\mathrm{^{\prime}}}\left(C\right)\cdot \left\{\left[{\Gamma }_{vv}\left({M}_{j}\right)-{\Gamma }_{vv}\left(C\right)\right]\right\},$$

where, for simplicity and in line with the sparse nature of SMAP-Reflectometry and the fact that ${\Gamma }_{HV }\sim {\Gamma }_{VH}$ are at least an order of magnitude smaller than ${\Gamma }_{HH}$ and ${\Gamma }_{VV}$ at L-band^40^, we consider $\zeta \cdot \left[{\Gamma }_{vh}\left(C\right)-{\Gamma }_{vh}\left({M}_{j}\right)\right]=0$. The coarse and medium data ${\Gamma }_{vv}\left(C\right)$ and ${\Gamma }_{vv}\left({M}_{j}\right)$ are obtained by aggregating SMAP-Reflectometry data into 36 km pixels and 9 km pixels, respectively, and then applying a natural interpolator to complete missing data.

**Figure 8:**
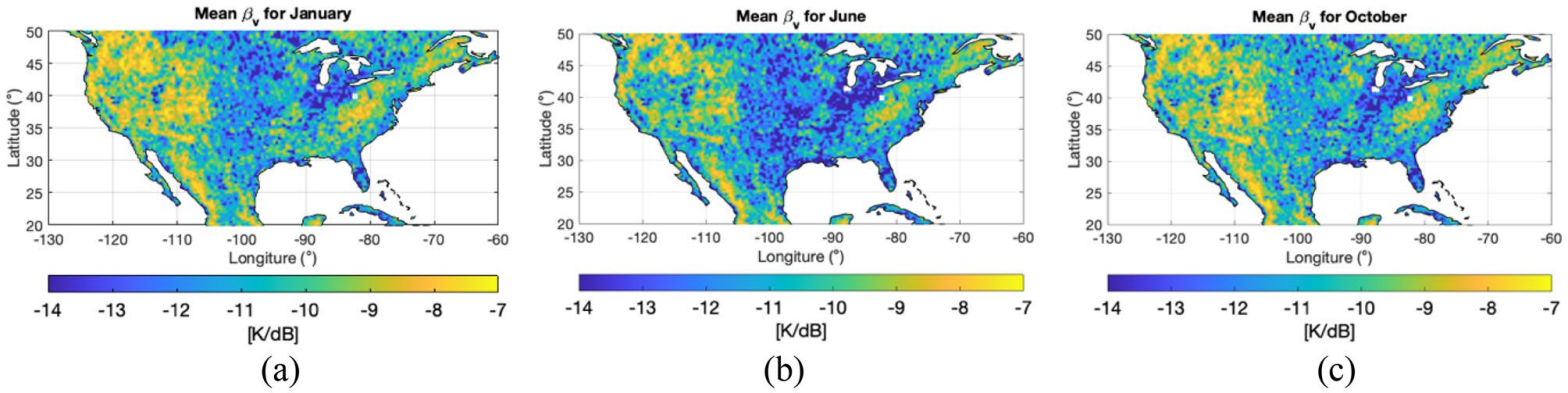
Monthly ${\beta }^{\mathrm{^{\prime}}}$ obtained for three different months over the CONUS region: (**a**) January, (**b**) June and (**c**) October, 2018. All figures made with Matlab R2021b (version 9.11.0.1809720, URL: https://www.mathworks.com/products/new_products/release2021b.html).

We then interpolate the 36 km ${T}_{Bv}$ data into a 9 km grid using Eq. (4) and ${\beta }^{\mathrm{^{\prime}}}$ montly maps, such as the ones in Fig. 8. Finally, we model the SM values provided in SMAP datasets. The model is obtained as a neural network with two hidden layers with seven neurons each and trained to reproduce the SM retrievals from SMAP datasets. We use a subset of the original ${T}_{Bv,h}$ and their associated ancillary datasets included in the SMAP L3 36 km^39^, i.e. land surface temperature and VOD, as inputs of the neural network. The target output of the neural network is selected as the SM values obtained by the mission’s single-channel algorithm at V polarization (SCA-V). Therefore, we build a neural network trained to produce very close output to the SMAP’s mission. Once trained, the neural network is validated against a different subset of original ${T}_{Bv,h}$, land surface temperature and VOD values. Our implementation shows an R^2^ = 0.989 and root-mean-square error of 0.021 m^3^/m^3^ with respect to the current SMAP SCA-V product. Once we have the neural network validated to mimic the SCA-V algorithm, we use it to compute SM values, i.e. the neural network is applied to both radiometer only 36 km ${T}_{Bv,h}$ and aided-interpolated ${T}_{Bv,h}$ at 9 km to produce the corresponding SM retrievals, making them comparable within our research.

## Conclusions

The capability of SMAP as the first fully polarimetric GNSS-R mission is retrieved from a dormant dataset, and its value to the SMAP mission and the GNSS-R community has been discussed. This work demonstrates the value of adding SMAP-Reflectometer polarimetric information to areas with high VOD values to improve the SM estimation of the SMAP mission. The effect of the vegetation on the SM estimation is better resolved when the GPS forward scattered signals are used in the retrieval because those signals are sensitive to the vegetation volume scattering and the surface soil moisture. As an independent source of information, SMAP-Reflectometer helps the SM estimations of the radiometer, which are based primarily on the spontaneous emission of the scene and the normalized difference vegetation index (NDVI) from multispectral sensors. As the vegetation dynamics are slower than the SM, especially over the forest and dense vegetation areas, the SMAP-Reflectometer data assessments over seasonal periods can reduce the error in the estimations of SMAP daily SM products. The polarimetric information of this unique GNSS-R dataset will also be important to other areas of study, such as the cryosphere, where polarimetric signatures will be key to further understanding sea ice characteristics or land F/T state transitions, for example. Consequently, additional studies will be conducted to increase the value of the SMAP-Reflectometer to the SMAP mission’s F/T product in roles similar to the one intended for the radar.

## Data Availability

The datasets generated and/or analyzed during the current study are not publicly available as those are planned to be released as an official SMAP mission dataset by the end of the current NASA ROSES funded project, but are available from the corresponding author on reasonable request.
